# Cross-Sectional Profiling of Aqueous Humor VEGF-A Reflects the Tumor and Ocular Microenvironment in Retinoblastoma

**DOI:** 10.1167/iovs.67.12.23

**Published:** 2026-10-14

**Authors:** Michelle Lin, Chen-Ching Peng, Mark Reid, Elaine Huang, Nerea Goni, Kaitlyn Phung, Yilin Chen, Drishti Pandya, Brianne Brown, Bibiana J. Reiser, Rachana Shah, Jesse L. Berry, Liya Xu

**Affiliations:** 1 The Vision Center, Children's Hospital Los Angeles, Los Angeles, California, United States; 2 USC Roski Eye Institute, University of Southern California Keck School of Medicine, Los Angeles, California, United States; 3 Division of Hematology Oncology, Cancer and Blood Disease Institute, Children's Hospital Los Angeles, Los Angeles, California, United States; 4 University of Southern California Keck School of Medicine, Los Angeles, California, United States

**Keywords:** retinoblastoma (RB), vascular endothelial growth factor A (VEGF-A), aqueous humor (AH)

## Abstract

**Purpose:**

Vascular endothelial growth factor A (VEGF-A) is a key mediator of tumor angiogenesis. This cross-sectional study quantified aqueous humor (AH) VEGF-A in retinoblastoma (RB) eyes and retrospectively evaluated its association with clinical features.

**Methods:**

This study analyzed 60 AH samples: 54 from 49 RB eyes and 6 from age-matched controls with congenital cataract. From pretreatment RB eyes, six samples were collected prior to globe-salvaging treatment and five immediately after primary enucleation (PE). From post-treatment eyes, 12 samples were collected at secondary enucleation (SE), 12 during active treatment (TX), and 19 at 6 months after treatment (END). VEGF-A was measured using the Olink Proximity Extension Assay and reported as absolute concentrations (pg/mL).

**Results:**

VEGF-A concentrations (median and interquartile range [IQR]) were 259 (95% confidence interval [CI] = 140–423, IQR = 115–568) in the RB cohort, compared with 149 (95% CI = 39.8–318, IQR = 61.3–208) in cataract controls. Post-treatment levels were significantly lower than pretreatment levels (median = 182, 95% CI = 121–310, IQR = 110–423 vs. median = 1380, 95% CI *=* 278–18,100, IQR = 467–12,400, *P* < 0.001). Among treated eyes, SE samples had higher VEGF-A than END samples (median = 506, 95% CI = 120–1010, IQR = 207–926 vs. median = 129, 95% CI = 79.5–212, IQR = 79.5–212, *P* = 0.007).

**Conclusions:**

AH liquid biopsy was a high-yield source of VEGF-A in RB. VEGF-A concentrations were highest during primary enucleation and normalized after treatment completion. These findings support that aqueous VEGF-A reflects the intraocular microenvironment, warranting further validation to improve our understanding of its role in disease progression in RB.

Retinoblastoma (RB) is a pediatric retinal tumor characterized by dysregulated angiogenic signaling, including increased expression of vascular endothelial growth factor A (VEGF-A).^1^ Until recently, molecular characterization of RB was largely limited to enucleated eyes due to the inability to directly biopsy the tumor without increasing iatrogenic risk of metastasis.^2^^–^^5^ However, the establishment of aqueous humor (AH) as a surrogate for direct tumor biopsy has opened new frontiers in RB, enabling in vivo biomarker discovery.^6^^–^^12^ Multiple AH diagnostic and prognostic biomarkers, such as tumor-derived nucleic acids, tumor methylation status, extracellular vesicles, and proteins, have been explored in patients undergoing globe-salvaging therapy to better understand their roles in RB tumor progression.^12^^,^^13^

VEGF-A is a key member of the VEGF family of proteins that promotes endothelial cell proliferation and migration, playing an essential role in physiologic and pathologic angiogenesis.^14^^,^^15^ In tumors, multiple triggers within the microenvironment can disrupt the critical balance between pro- and anti-angiogenic factors, tipping the scale toward pathologic angiogenesis. These triggers include genetic alterations, trauma, oxidative stress, mechanical strain, and hypoxia, which is known to be a particularly potent inducer.^15^^–^^18^ Synergistic treatment combining anti-VEGF therapy with chemotherapy has demonstrated clinical efficacy in colorectal cancer,^19^^,^^20^ non-small cell lung cancer,^21^ renal cell carcinoma,^22^^,^^23^ hepatocellular carcinoma,^24^ glioblastoma multiforme,^25^ ovarian cancer,^26^^,^^27^ and cervical cancer,^28^ leading to regulatory approvals in the United States and in other countries. Additionally, intravitreal delivery of an anti-VEGF agent, bevacizumab, is standard of care in multiple ocular conditions,^29^ such as proliferative diabetic retinopathy, neovascular age-related macular degeneration, and macular edema due to retinal vein occlusion.

In RB, VEGF-A is proposed to facilitate disease progression by supporting vascular stroma formation and nutrient delivery to the tumor.^30^ Earlier studies demonstrated VEGF-A mRNA overexpression in paraffin-embedded RB tumor sections from enucleated eyes, as well as VEGF-A upregulation in vitreous samples from two patients with aggressive bilateral RB.^30^^,^^31^ Additionally, higher microvascular densities and vascular area, measured via immunohistochemistry, have been correlated with aggressive tumor behavior and metastasis.^32^^,^^33^ More recently, the AH has emerged as a platform for VEGF-A analyses through liquid biopsy. Because the AH is protein-sparse under normal conditions, monitoring protein concentrations throughout active therapy offers promising potential for the discovery of novel protein biomarkers in RB.^13^^,^^34^^,^^35^ In 2017, Cheng and colleagues were the first to report significantly higher RB AH VEGF-A concentrations among 45 cytokines/chemokines, matrix metalloproteinases, and acute phase proteins of interest.^14^ Recently, Daniels and colleagues used the enzyme-linked immunosorbent assay (ELISA) to measure VEGF-A concentrations in AH samples from five RB eyes receiving intravitreal chemotherapy injections with melphalan, and discovered that VEGF-A levels corresponded to clinical response patterns in longitudinal samples.^36^ Whereas previous studies have explored intraocular VEGF-A in RB, reporting of absolute AH VEGF-A concentrations across the timeline of RB diagnosis and treatment are limited.

Herein, we investigate the range of VEGF-A concentrations in a large cohort across multiple treatment timepoints and explore its relationship with clinical features and outcomes. We also establish the reliability of proximity extension assay (PEA) technology, a highly sensitive multiplexing platform, to quantify VEGF-A in AH. With these developments, this study delineates cross-sectional variations of VEGF-A throughout key clinical timepoints and provides a foundation to further our understanding of the protein's potential role for informing prognosis and clinical decision making in this rare pediatric intraocular cancer.

## Methods

This investigation was conducted with Institutional Review Board (IRB) approval at Children's Hospital Los Angeles (CHLA), and adhered to the tenets of the Declaration of Helsinki and the United States Health Insurance Portability and Accountability Act. Written informed consent for a biorepository was obtained from the parents or legal guardians of all patients. AH data were maintained separately from clinical data until final retrospective analysis.

### Sample Collection and Storage

The following procedure has been established in detail.^8^ A clear corneal paracentesis was performed to extract up to 0.1 mL of AH as part of routine procedure for anterior segment surgery for congenital cataract. All RB AH analyzed herein was residual specimen previously collected as part of an IRB-approved study investigating aqueous-derived molecular biomarkers to guide targeted, patient-centered therapies in RB. AH was collected at the time of diagnosis (DX), alongside globe-salvaging treatment (TX; at the end of systemic chemotherapy or as part of the procedure for intravitreal injection of chemotherapy^6^), at least 6 months after the completion of treatment (END; with no clinical evidence of active intraocular RB tumor or seeding), or immediately following enucleation (primary or secondary [PE] or [SE]). No AH was collected solely for VEGF-A analysis, and no adverse events occurred. The safety of AH liquid biopsy has been reported previously.^37^ AH sampled at any time other than during the procedure for intravitreal injection of chemotherapy was not part of routine clinical examination and was performed for research purposes as a designated timepoint under the CHLA IRB. Treatment of all patients with RB was performed in a non-randomized manner according to CHLA protocol,^38^^–^^40^ and treating physicians were blinded to the AH results. AH samples were stored at −80°C and thawed on ice immediately prior to analysis.

### Proximity Extension Assay for VEGF-A Quantification

VEGF-A was quantified in 5 µL AH samples using next-generation sequencing (NGS)-based PEA (Olink Proteomics, Uppsala, Sweden). Results were reported as normalized protein expression (NPX) values, which are log_2_-transformed, relative units that allow for comparison of relative protein abundance across samples analyzed within the same batch. Although NPX values are reproducible for relative comparisons, they do not reflect absolute protein concentrations and cannot be directly compared across experiments or clinical settings. To measure absolute quantification (AQ), 5 µL AH samples were submitted from a subset of our cohort (*n* = 7) for analysis as part of a larger batch of samples from different ocular diagnoses (total *n* = 38) via a qPCR-based PEA platform (Olink Proteomics). Results were reported as pg/mL. Data quality was maintained with Olink's built-in system using internal controls for each sample.^41^^,^^42^ According to the manufacturer's specifications, the mean intra-assay and inter-assay coefficients of variation for VEGF-A were both ≤7%. Samples with a deviation of ± 0.3 NPX from the median of all samples on the plate were flagged with a quality control warning, and only those within the upper and lower limits of detection were included in the final analysis. PEA results were validated with ELISA (see Supplementary Material).

### Bootstrapped Cross-Validated Linear Model

A linear regression model was used to assess the relationship between 38 known NPX and AQ values (see Supplementary Material). We performed a bootstrap procedure with 1000 iterations, sampling 20 pairs of NPX and AQ values with replacement each time. The median slope, intercept, *P* value, and *R*^2^ from this procedure were nearly identical to those from the original 38-sample linear regression. Because our goal is to base downstream comparisons on the observed data rather than the bootstrapped resamples, the original regression results have been reported herein. Bridging samples were included to correct for batch effects prior to model fitting.

### Statistical Analysis

In this analysis, RB eyes were categorized into two groups based on treatment exposure: pretreatment eyes (AH obtained prior to initiation of therapy) and post-treatment eyes (AH obtained after any therapeutic intervention). Pretreatment eyes include AH samples labeled as DX or PE; and post-treatment eyes include AH samples labeled as TX, END, or SE. AH from patients with congenital cataract served as controls. Given glaucoma is known to cause elevations in VEGF-A, eyes with congenital glaucoma were excluded from analysis.

VEGF-A concentrations are reported in pg/mL and continuous variables are summarized as the median with the 95% confidence interval (CI), interquartile range (IQR), and range, unless otherwise specified. Nonparametric Mann-Whitney *U* tests were used to compare non-normally distributed VEGF-A concentrations by clinical diagnosis, clinical outcome, treatment status, sex, *RB1* mutation, and laterality. Spearman's correlation was used to assess the association between VEGF-A concentrations and age at diagnosis (months). Multiple regression models with Huber-White robust standard errors were used to compare VEGF-A concentrations (ranked) by clinical outcome and treatment status while controlling for previous treatment exposure (any use of radiation; number of sessions of intra-arterial chemotherapy [IAC], cryotherapy, and laser photocoagulation) and disease characteristics (any iris neovascularization) that may impact VEGF-A. All *P* values less than 0.05 were considered statistically significant. Statistical analyses and plots were performed using GraphPad Prism version 10.0.3 (GraphPad Software, Boston, MA, USA).

## Results

### Participant Characteristics

A total of 60 AH samples were analyzed, including 54 samples from 49 eyes of 44 patients with RB, and 6 samples from 5 age-matched patients with congenital cataract serving as controls (see Fig. 1; Supplementary Fig. S1). Eleven AH samples were collected from pretreatment RB eyes, including six diagnostic and five PE samples. Forty-three AH samples were collected from post-treatment RB eyes, including 12 SE, 12 active treatment (TX), and 19 end-of-treatment (END) samples. Nineteen (43%) female patients and 25 (57%) male patients were included, 29 (66%) with unilateral and 15 (34%) with bilateral disease. Median age of diagnosis was 13 months (range = 2–48 months). The majority of eyes were IIRC group D (69%) and American Joint Commission on Cancer (AJCC) clinical stage cT2B (82%). Five (10%) eyes had iris neovascularization (NVI), 4 (8%) had high intraocular pressure (IOP), 2 (4%) had neovascular glaucoma, and 4 (8%) had anterior segment involvement (see Supplementary Data Excel File). Among the 21 enucleated eyes, 4 (19%) had NVI at the time of AH sample collection. Among post-treatment eyes, 36 (90%) were treated with laser photocoagulation, 7 (18%) with cryotherapy, 11 (28%) with IAC, and 4 (10%) with radiation. Of the 60 samples, 7 were directly measured AQ values and 53 were AQ values predicted from NPX using the validated linear model (see Supplementary Fig. S2).

**Figure 1.**
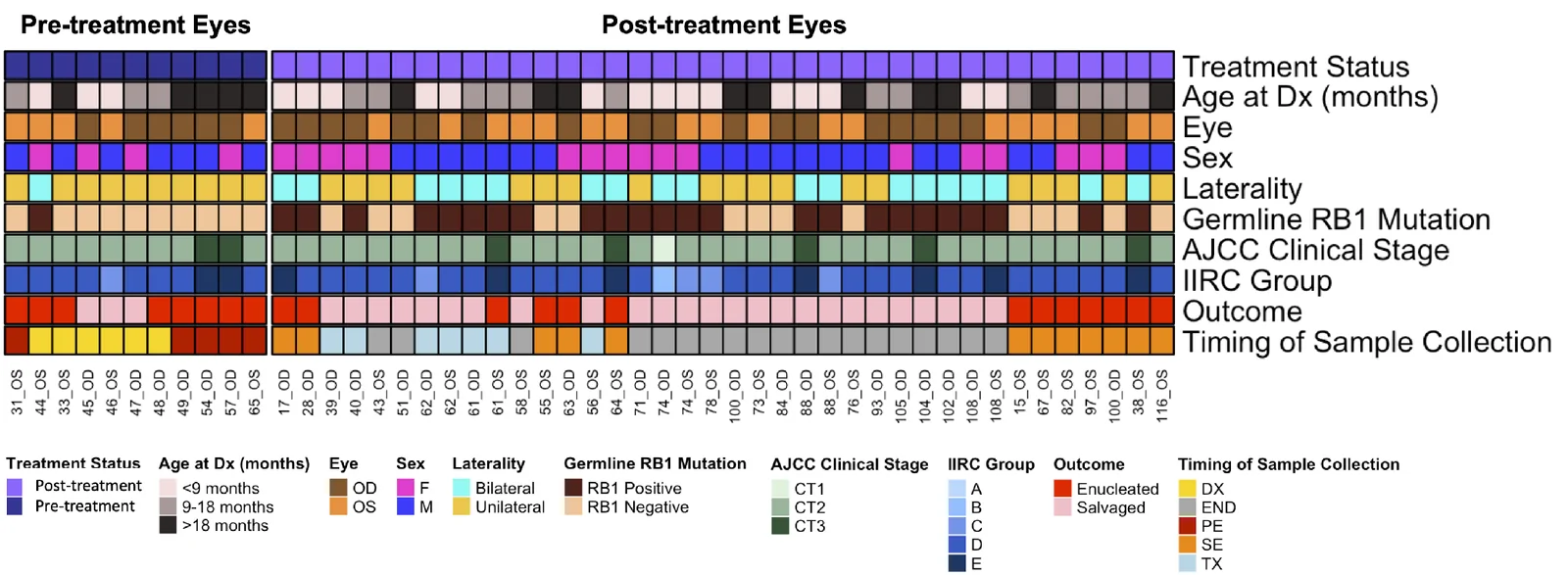
Clinical and demographic features for each RB eye included in analysis, organized by treatment status. DX, diagnosis; END, end of treatment; PE, primary enucleation; SE, secondary enucleation; TX, active treatment. Cases 33_OS, 40_OD, 44_OS, and 61_OD contributed two or more aqueous humor samples. Please refer to the Supplementary Data Excel File for further information.

### VEGF-A Is Elevated in Retinoblastoma Compared to Non-Tumor Pediatric Eyes

VEGF-A concentrations were elevated in RB AH compared to non-tumor controls. Median VEGF-A concentrations were low in congenital cataract AH 149 (95% CI = 39.8–318, IQR = 61.3–208, range = 39.8–318; see Fig. 2). In the RB cohort, VEGF-A levels were highly variable 259 (95% CI = 140–423, IQR = 115–568, range = 54.0–18,100), demonstrating that VEGF-A is biologically active in the RB tumor microenvironment.

**Figure 2.**
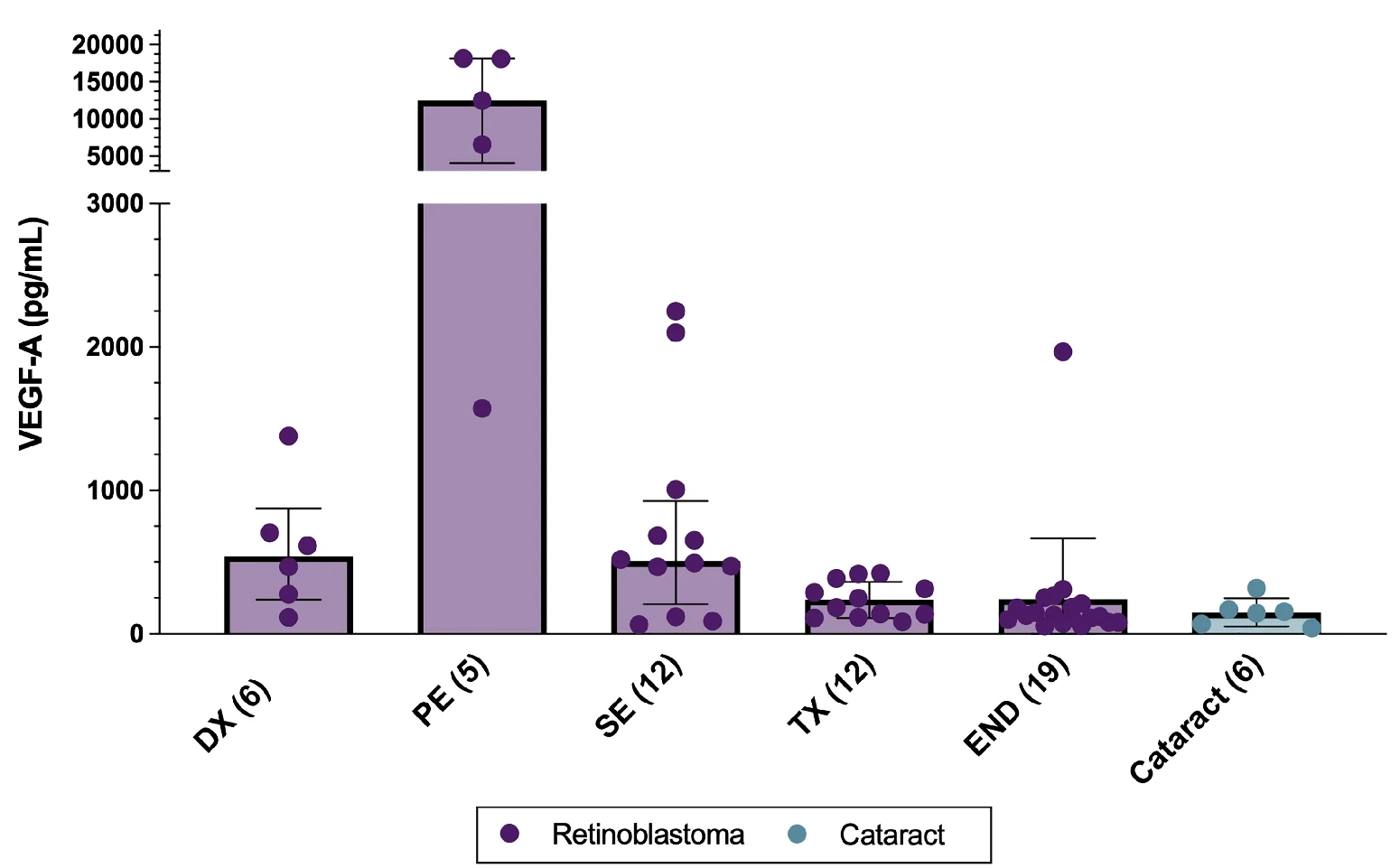
Quantitative comparison of median aqueous humor (AH) VEGF-A concentrations among RB and cataract eyes. Among RB eyes, AH samples were collected at diagnosis (DX), primary enucleation (PE), secondary enucleation (SE), active treatment (TX), or end of treatment (END). Error bars represent interquartile range.

### Impact of Treatment on VEGF-A Levels

When RB eyes were grouped by treatment exposure, VEGF-A concentrations were significantly lower in AH from post-treatment eyes compared with pretreatment eyes (182, 95% CI = 121–310, IQR = 110–423, range = 54.0–2250 vs. 1380, 95% CI = 278–18,100, IQR = 467–12,400, range = 115–18,100, *P* < 0.001; see Fig. 3A). This association remained significant in a regression analysis controlling for covariates, including NVI, radiation treatment, and number of sessions of IAC, cryotherapy, and laser photocoagulation (β = −10.92, 95% CI = −18.89 to −2.95, *P* = 0.007). VEGF-A levels were highest in samples collected at the time of primary enucleation (*n* = 5, range = 1570–18,100), and lowest at the END (129, 95% CI = 79.5–212, IQR = 79.5–212, range = 54.0–1970; see Fig. 2). Altogether, VEGF-A concentrations varied widely across clinical treatment timepoints, with higher levels observed in eyes prior to initiation of any therapy.

**Figure 3.**
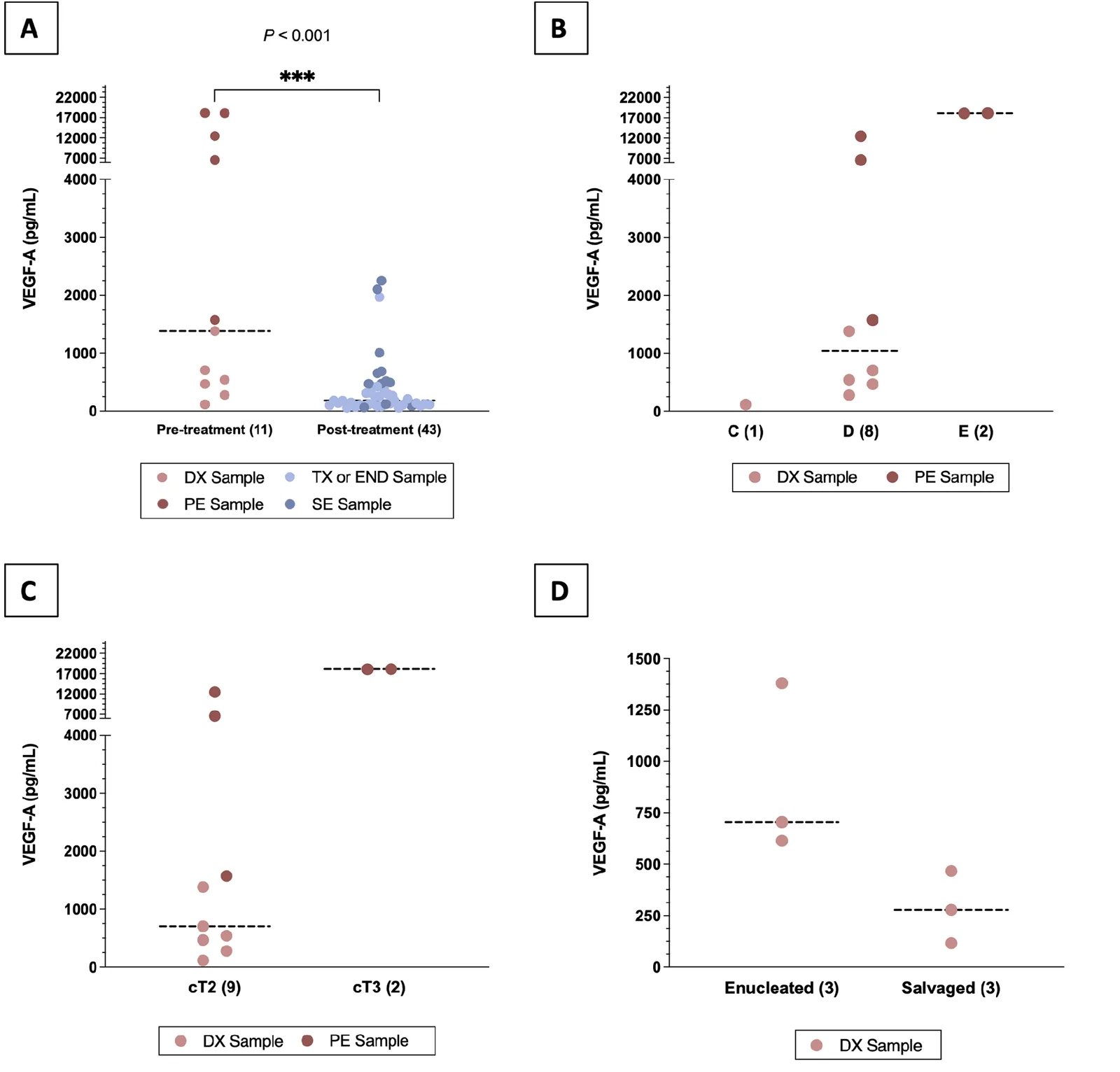
Quantification of VEGF-A in AH samples collected from pretreatment and post-treatment eyes. (**A**) Overall comparison of median VEGF-A concentrations between samples taken from pretreatment and post-treatment eyes. *P* value displayed on the graph is derived from the Mann-Whitney *U* test; the association remained significant following adjustment for previous treatment exposure and disease characteristics (*P* = 0.007). (**B**) VEGF-A concentrations stratified by IIRC group in pretreatment eyes. (**C**) VEGF-A concentrations stratified by AJCC clinical stage in pre-treatment eyes. (**D**) Comparison of VEGF-A concentrations by clinical outcome (enucleation versus salvaged) from six AH samples taken at the time of RB diagnosis. ***P < 0.001.

### Magnitude of VEGF-A Elevation by Tumor Stage at Diagnosis

We further describe VEGF-A concentrations across tumor classification subgroups from pretreatment eyes by the International Intraocular Retinoblastoma Classification (IIRC; groups A–E) and AJCC clinical staging systems. By IIRC tumor classification, VEGF-A concentrations were 115 in a single group C eye, 1040, 95% CI = 278–12,500, IQR = 485–5330, range = 278–12,500 for group D eyes (*n* = 8), and 18,150 and 18,110 for group E eyes (*n* = 2; see Fig. 3B). By AJCC clinical staging, VEGF-A concentrations were 705 (95% CI = 278–6590, IQR = 372–4080, range = 115–12,500) in cT2 eyes (*n* = 9), and 18,150 and 18,110 in cT3 eyes (*n* = 2; (see Fig. 3C). One group D/cT2 eye was reported to have anterior segment involvement due to tumor adjacent to the pars plana and elevated IOP, however, it was unclear whether the tumor was anterior to the vitreous face; the two group E/cT3 eyes were reported to have NVI, high IOP, and neovascular glaucoma. All three of these eyes underwent PE.

### Elevated VEGF-A Is Associated With Treatment Failure and Enucleation

Among the six RB eyes with AH samples collected at the time of diagnosis, three eyes were salvaged and three underwent SE following standard therapy. VEGF-A concentrations at diagnosis appeared higher in eyes that were later enucleated (*n* = 3: 1380, 705, and 614) compared with eyes that were salvaged (*n* = 3: 467, 278, and 115; see Fig. 3D), suggesting higher levels at diagnosis may be associated with advanced disease.

When AH from post-treatment RB eyes were organized by outcome (SE versus END), the END samples exhibited significantly lower VEGF-A concentrations compared with SE samples (*P* = 0.007; see Fig. 4A). END samples had a median value of 129 (95% CI = 79.5–212, IQR = 79.5–212, range = 54,0–1970) and SE samples had a median value of 506 (95% CI = 120–1010, IQR = 207–926, range = 64.7–2250). This association remained significant after accounting for previous treatment exposure and disease characteristics as previously described (β = −13.94, 95% CI = −23.54 to −4.33, *P* = 0.004). Moreover, VEGF-A concentrations in END samples were comparable to those observed in congenital cataract controls 149 (95% CI = 39.8–318, IQR = 61.3–208, range = 39.8–318; see Fig. 4B). Notably, one END sample from an eye previously treated with external beam radiation therapy (EBRT) showed persistently elevated VEGF-A levels compared to the remaining END cohort. This eye did not have NVI or neovascular glaucoma. Overall, these patterns suggest that VEGF-A levels normalize after the completion of salvage therapy, and low VEGF-A levels in this group may indicate tumor quiescence.

**Figure 4.**
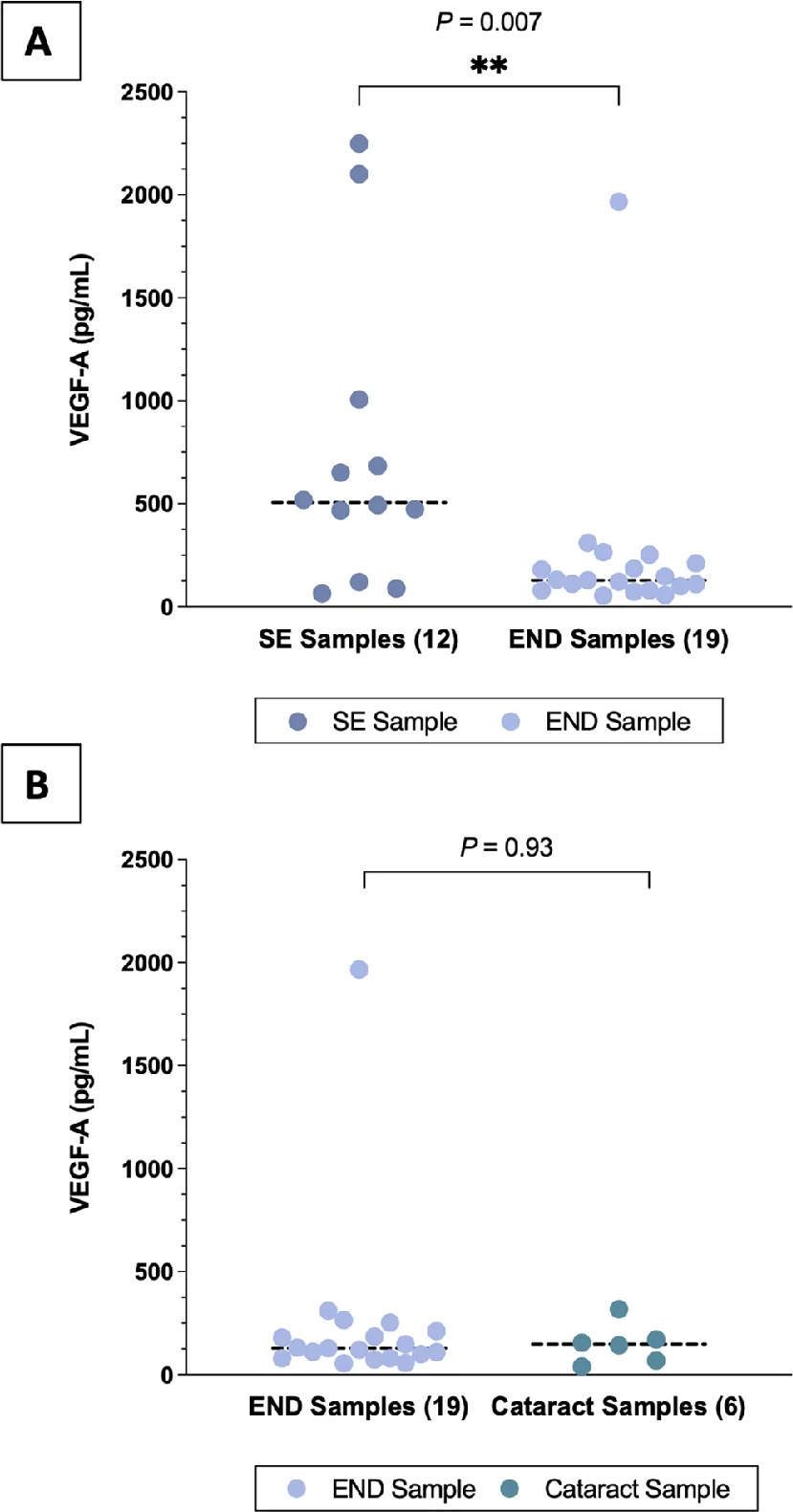
Median VEGF-A concentrations in treated RB eyes, compared (**A**) between secondarily enucleated and end of treatment samples, and (**B**) between end of treatment and cataract samples. *P* values displayed on the graph are derived from the Mann-Whitney *U* test; the association in **A** remained significant following multivariate adjustment for previous treatment exposure and disease characteristics (*P* = 0.004). SE, secondary enucleation; **P < 0.01.

## Discussion

Our study included the largest RB cohort thus far, to our knowledge, quantifying VEGF-A in AH from multiple timepoints of treatment, including at DX, to represent the full spectrum of disease. Although ELISA has been previously used to quantify AH VEGF-A, its requirement for large sample volumes limits its application in rare pediatric tumors like RB. To address this, we utilized PEA technology for highly sensitive protein quantification requiring minimal sample input, and demonstrated cross-method agreement between ELISA and PEA to reinforce use of PEA in AH protein analysis for RB (see Supplementary Table S1). With a total of 60 AH samples, we found that VEGF-A concentrations were variable in RB compared with congenital cataract eyes. In RB eyes, VEGF-A levels were differentiated by treatment status and clinical outcomes, which remained significant after adjusting for covariates such as iris neovascularization, radiation, intra-arterial chemotherapy, laser photocoagulation, and cryotherapy. AH sampled at the time of PE harbored the highest VEGF-A levels, whereas those at the end of globe-conserving therapy demonstrated the lowest levels. In a small number of AH samples, VEGF-A levels measured at the time of DX trended higher in eyes that ultimately required enucleation compared with those that were salvaged.

In disease-free states, AH is relatively protein-sparse, making it a particularly promising biofluid for proteomic biomarker discovery in RB and can be done with minute volumes of AH.^43^ Using gel electrophoresis in 18 RB eyes undergoing enucleation, Hadjistilianou and colleagues reported elevated AH protein content in RB eyes compared with controls, and the highest levels in eyes with secondary glaucoma.^44^ Recently, Daniels and colleagues proposed for the first time that aqueous VEGF-A may serve as a molecular biomarker of treatment response in RB.^36^ With longitudinal samples from five patients undergoing intravitreal melphalan injections for vitreous seeding, they established a correlation between VEGF-A levels and vitreous seed response to treatment (clinical progression, regression, or stability), establishing the feasibility of in vivo quantification of VEGF-A in RB AH.^36^ Building from their findings, our analysis revealed wide variability of VEGF-A concentrations in RB compared with non-tumor pediatric controls, which may have reflected the sensitivity of PEA technology and the inclusion of AH samples from diverse timepoints of treatment. These exploratory findings support RB as a VEGF-A-active disease, allowing for further investigation into VEGF-A's role in disease progression and its potential as an intraocular biomarker of treatment response in RB.

When RB eyes were grouped by exposure to therapy, we found significantly higher VEGF-A concentrations in pretreatment compared with post-treatment eyes, which is consistent with previous studies showing decreased protein content following therapy.^36^^,^^44^ In the pretreatment group, VEGF-A concentrations were greatest in eyes with advanced tumor stages and in those requiring PE (*n* = 5), of which two were IIRC group E eyes with iris neovascularization and neovascular glaucoma, and one was an IIRC group D eye with likely anterior segment tumor involvement and elevated IOP. Yet, after controlling for these additional clinical variables (e.g. treatment with radiation, intra-arterial chemotherapy, laser photocoagulation, and cryotherapy), the median pretreatment concentration remained significantly greater than that of the post-treatment group. This finding demonstrated a distinction between treatment-naïve and treatment-exposed intraocular environments, driven by notable elevations in eyes that underwent PE. Because VEGF-A is a nonspecific protein, its levels likely reflect a complex intraocular milieu influenced by the presence of tumor activity, retinal tissue ischemia, inflammation, and recent treatments.^45^^,^^46^

In our study, the post-treatment group was comprised of aqueous collected from multiple therapy timepoints: active treatment, 6 months after the end of treatment, and at the time of SE. To compare VEGF-A patterns in this heterogeneous cohort, we measured END levels in successfully salvaged eyes versus secondarily enucleated eyes, and found that the median VEGF-A was significantly lower in salvaged eyes, which was comparable to that of cataract eyes. However, two eyes in the END cohort received radiation treatment, one with plaque brachytherapy (case 43_OS) and the other with external beam radiation therapy (case 102_OD), the latter which harbored a VEGF-A concentration over 15-fold greater than the median concentration of the entire END cohort. Therefore, whereas low VEGF-A levels were observed at the completion of treatment, these should be evaluated in the overall clinical context to account for multiple origins of intraocular VEGF-A, such as subretinal fluid, retinal detachment, or treatment-related vascular compromise. RNAscope technology is one way to distinguish between neoplastic and ischemic sources of VEGF-A, as demonstrated in a study that used a rabbit RB model to report high *VEGF-A* mRNA expression in vitreous seeds compared to surrounding retinal tissue.^36^ This study would be difficult to replicate in a salvaged human RB eye due to our inability to directly biopsy the tumor, therefore, assessing the magnitude of tumor-derived VEGF-A relative to other sources of VEGF-A requires further investigation.

A limitation of our study was the lack of sequential AH samples among salvaged eyes. Therefore, we were unable to determine whether VEGF-A changes within individual eyes over time, and future studies should consider longitudinal measurements in eyes undergoing treatment. An additional limitation was the prediction of AH VEGF-A concentrations from NPX using a linear model; thus we reported a dynamic range rather than direct measurements. However, bootstrapping analyses were used to validate the model prior to its application. Last, we used multiple Olink PEA batches which introduced inter-run variability, and applied batch correction procedures across datasets to address this as per standard protocol for proteomic studies.

## Conclusions

Our study identified AH liquid biopsy as a high-yield source of VEGF-A in RB at diagnosis and across multiple treatment timepoints. VEGF-A levels were elevated in eyes prior to the initiation of therapy, and the highest levels were associated with eyes undergoing PE. Among treated eyes, VEGF-A levels returned to a physiologic baseline in those that completed globe-conserving therapy. Because VEGF-A is a nonspecific protein, intraocular concentrations are influenced by multiple processes and reflects a composite signal of both the tumor and ocular microenvironment. Prospective, serial measurements of VEGF-A within larger RB cohorts will be essential to better understand how VEGF-A responds to therapy, and to further explore the role of AH liquid biopsy as a monitoring strategy throughout the timeline of personalized treatment and clinical decision making in RB.

## Supplementary Material

Supplement 1

Supplement 2
