# Supplementary material for: Cross-Sectional Profiling of Aqueous Humor VEGF-A Reflects the Tumor and Ocular Microenvironment in Retinoblastoma
Source: Invest Ophthalmol Vis Sci. 2026 Oct 14;67(12):23. doi: 10.1167/iovs.67.12.23 (PMC13676253; doi:10.1167/iovs.67.12.23)
Supplement: Supplementary file 1 [file iovs-67-12-23_s001.docx]

**Supplementary Material**

**Table of Contents**

| **Content** | **Page** |
| --- | --- |
| **Supplementary Methods**  *Quantification of AH VEGF-A using ELISA* | 2 |
| **Supplementary Results**  *ELISA Confirms Elevated VEGF-A in Congenital Glaucoma AH*  *Validation of PEA with ELISA*  *Linear Regression Model* | 2  3  3 |
| **Tables**  Supplementary Table S1 | 4 |
| **Figures**  Supplementary Figure S1  Supplementary Figure S2 | 5  6 |

**Supplementary Methods**

*Quantification of Aqueous Humor VEGF-A using the Enzyme-linked Immunosorbent Assay (ELISA)*

In order to validate the proximity extension assay (PEA) with ELISA, aqueous humor (AH) VEGF-A was quantified in an eye with congenital glaucoma with the ELISA platform. Glaucoma was chosen because previous literature has reported VEGF-A elevation in the AH from eyes with different types of glaucoma.^1-6^ This sample was used solely for cross-platform comparison and not for biological interpretation or control comparison. The sample was analyzed using a DuoSet ELISA for hVEGF-A (R&D Systems, DY293B-05) according to manufacturer-provided instructions. Briefly, 10 μL of AH was diluted in 90 μL of sample diluent (total 100 μL).

**Supplementary Results**

*ELISA Confirms Elevated VEGF-A in Congenital Glaucoma Aqueous Humor*

Using ELISA, we quantified AH VEGF-A from an eye with congenital glaucoma, revealing a concentration of 609 pg/mL. This concentration was higher than VEGF-A levels reported in a recent study by Daniels and colleagues **(**see **Supplementary Table S1)**. Previous studies have reported measuring VEGF or VEGF-A in AH from eyes with different types of glaucoma, including neovascular glaucoma, primary open angle glaucoma, and acute angle glaucoma, and demonstrated wide-ranging concentrations **(**see **Supplementary Table S1**).

*Validation of Proximity Extension Assay (PEA) with ELISA*

AH VEGF-A was measured from the same eye with congenital glaucoma using the Olink PEA platform (Olink® Proteomics, Uppsala, Sweden). The final readout was reported as Normalized Protein Expression (NPX) (arbitrary units on a log_2_ scale) for relative quantification and as pg/mL for absolute quantification (AQ). We detected a mean concentration of 670.2 ± 174.6 pg/mL, which was comparable to our ELISA concentration of 609 pg/mL. The measurements demonstrated close agreement, supporting consistency between the two technologies. While this comparison is limited to a single sample, it provides preliminary support for cross-platform consistency.

*Linear Regression Model*

A linear model on the log_2_ scale was fit on 38 AH samples with both known normalized protein expression (NPX) and AQ values (see **Supplementary Figure S2**). The 38 samples represented different ocular diseases including retinoblastoma, cataract, glaucoma, pediatric retinal diseases, and uveal melanoma, and were not limited to those analyzed in this study. The model was log_2_ (pg/mL) = 7.51 + 1.02×NPX (slope 1.024; 95% CI: 0.939–1.110; *R*^2^ = 0.94; RMSE = 0.42 log_2_), with a significant overall fit (*F* (1, 36) = 589.3, *P* < 0.0001). This model was applied to predict absolute concentrations for 53 samples in our study with only NPX values available. Across a total of 60 AH samples in our cohort, 7 were directly measured AQ values and 53 were AQ values predicted using this calibration model.

**Supplementary Table S1.** Comparison of aqueous humor VEGF-A quantification in glaucoma eyes from the literature with those using ELISA and PEA technologies in our study.

| Study | Sample Size | Disease | | Protein Target | Detection Method | Concentration (Mean ± SD, pg/mL) |
| --- | --- | --- | --- | --- | --- | --- |
| Our study | 1 | Congenital glaucoma | | VEGF-A | ELISA | 609.4 |
| Our study | 1 | Congenital glaucoma | | VEGF-A | PEA | 670.2 ± 174.6 |
| Daniels et al. 2024 | 2 | Congenital glaucoma | | VEGF-A | ELISA | < 60 |
| Nada and Abdel-Moety 2017 | 30 | NVG | VEGF | | ELISA | 832.88 ± 96.44 |
| Tripathi et al. 1998 | 12 NVG  15 POAG | NVG; POAG | | VEGF | ELISA | NVG (Mean ± SEM): 29267 ± 7350;  POAG (Mean ± SEM):  726 ± 204 |
| Dimtsas et al. 2024 | 30 | POAG | | VEGF-A | Bead-based immunoassay | 166.37 ± 110.04 |
| Hu et al. 2002 | 54 | POAG;  ACG; XFG | | VEGF | ELISA | 146.7 ± 51.8 |
| Huang et al. 2016 | 36 PACG  38 APCG | PACG; APCG; | | VEGF | ELISA | PACG (Mean ± SE):  165 ± 37.5;  APCG (Mean ± SE):  935 ± 258 |

Abbreviations: VEGF-A, vascular endothelial growth factor A; ELISA, enzyme-linked immunosorbent assay; PEA, proximity extension assay; NVG, neovascular glaucoma; VEGF, vascular endothelial growth factor; POAG, primary open angle glaucoma; ACG, angle closure glaucoma; XFG, exfoliative glaucoma; PACG, primary angle closure glaucoma; APCG, acute primary closure glaucoma.

**Supplementary Figure S1:** Consort Diagram of all sixty aqueous humor samples collected from retinoblastoma and congenital cataract eyes.

Eye(s)

Aqueous Humor

Patient(s)

Diagnosis

VEGF-A AHLB

RB

44

49

Diagnostic (DX) 6

Primary Enucleation (PE) 5

Active Treatment (TX) 12

Secondary Enucleation (SE) 12

End of Treatment (END) 19

Congenital Cataract

5

6

6

Abbreviations: VEGF-A, vascular endothelial growth factor-A; AHLB, aqueous humor liquid biopsy; RB, retinoblastoma.

**Supplementary Figure S2:** Calibration of normalized protein expression (NPX) to absolute quantification (AQ) values using a linear regression model with bootstrap resampling. Thirty-eight known absolute quantification values from different ocular diseases were included in the model to predict absolute values for 53 retinoblastoma samples. These predictions were used to estimate dynamic range rather than to infer precise absolute concentrations.

**References**

1.	Daniels AB, Sishtla KL, Bogan CM, et al. Aqueous VEGF-A Levels as a Liquid Biopsy Biomarker of Retinoblastoma Vitreous Seed Response to Therapy. *Invest Ophthalmol Vis Sci*. Jun 03 2024;65(6):18. doi:10.1167/iovs.65.6.18

2.	Nada WM, Abdel-Moety DA. Evaluation of Serum and Aqueous Humor Vascular Endothelial Growth Factor in Neovascular Glaucoma. Open Journal of Ophthalmology2017. p. 79-87.

3.	Tripathi RC, Li J, Tripathi BJ, Chalam KV, Adamis AP. Increased level of vascular endothelial growth factor in aqueous humor of patients with neovascular glaucoma. *Ophthalmology*. Feb 1998;105(2):232-7. doi:10.1016/s0161-6420(98)92782-8

4.	Dimtsas GS, Ieronymaki A, Chatzistefanou KI, Siasos G, Krassas A, Moschos MM. Elevated VEGF-A Levels in the Aqueous Humor of Patients With Primary Open Angle Glaucoma. *In Vivo*. 2024;38(4):1875-1881. doi:10.21873/invivo.13642

5.	Hu DN, Ritch R, Liebmann J, Liu Y, Cheng B, Hu MS. Vascular endothelial growth factor is increased in aqueous humor of glaucomatous eyes. *J Glaucoma*. Oct 2002;11(5):406-10. doi:10.1097/00061198-200210000-00006

6.	Huang W, Gao X, Chen S, Li X, Zhang X. Vascular Endothelial Growth Factor is Increased in Aqueous Humor of Acute Primary Angle-Closure Eyes. *J Glaucoma*. Jul 2016;25(7):e647-51. doi:10.1097/IJG.0000000000000242
